# Structural Formation and Photocatalytic Activity of Magnetron Sputtered Titania and Doped-Titania Coatings

**DOI:** 10.3390/molecules191016327

**Published:** 2014-10-13

**Authors:** Peter J. Kelly, Glen T. West, Marina Ratova, Leanne Fisher, Soheyla Ostovarpour, Joanna Verran

**Affiliations:** 1 Surface Engineering Group, Dalton Research Institute, Manchester Metropolitan University, Manchester M1 5GD, UK; E-Mails: g.west@mmu.ac.uk (G.T.W.); soheyla.ostovarpour@stu.mmu.ac.uk (S.O.); 2 School of Chemistry and Chemical Engineering, Queen’s University Belfast, Belfast BT9 5AG, UK; E-Mail: marina_ratova@hotmail.com; 3 School of Healthcare Science, Manchester Metropolitan University, Manchester M1 5GD, UK; E-Mails: l.fisher@mmu.ac.uk (L.F.); j.verran@mmu.ac.uk (J.V.)

**Keywords:** titanium dioxide, photocatalytic coatings, magnetron sputtering, doping, methylene blue, HiPIMS, antimicrobial activity

## Abstract

Titania and doped-titania coatings can be deposited by a wide range of techniques; this paper will concentrate on magnetron sputtering techniques, including “conventional” reactive co-sputtering from multiple metal targets and the recently introduced high power impulse magnetron sputtering (HiPIMS). The latter has been shown to deliver a relatively low thermal flux to the substrate, whilst still allowing the direct deposition of crystalline titania coatings and, therefore, offers the potential to deposit photocatalytically active titania coatings directly onto thermally sensitive substrates. The deposition of coatings via these techniques will be discussed, as will the characterisation of the coatings by XRD, SEM, EDX, optical spectroscopy, *etc.* The assessment of photocatalytic activity and photoactivity through the decomposition of an organic dye (methylene blue), the inactivation of *E. coli* microorganisms and the measurement of water contact angles will be described. The impact of different deposition technologies, doping and co-doping strategies on coating structure and activity will be also considered.

## 1. Introduction

Photocatalytic titania-based surfaces and coatings have many potential applications, including “self-cleaning” windows, anti-fogging screens or lenses, air cleaning and water purification devices and “self-sterilizing” antibacterial tiles [1,2,3,4,5,6]. Although it is relatively straightforward to demonstrate the effectiveness of these coatings in a laboratory environment, producing highly photoactive coatings in a commercially viable process is more challenging, and this has limited the exploitation of this technology to date. Titania can be produced in nanoparticle form for incorporation into paints and other building products [7], or as slurries and suspensions for water treatment [8,9]. Whilst the latter arrangement provides high surface areas of active material, there is usually a requirement for downstream filtration of the particles, limiting its practicality. In other applications, such as windows, lenses or tiles, a titania thin film or coating is the preferred option, where the reduced surface area is compensated for by high transparency and durability.

There are a number of physical and chemical deposition techniques that can be used to produce titania and doped-titania coatings. These include pulsed laser deposition [10], magnetron sputtering [11,12,13], reactive evaporation [13], ion beam assisted deposition [14], chemical vapour deposition [15], sol-gel [16], dip-coating [17], hydrothermal synthesis [18] and atomic layer deposition [19,20]. The characteristics of each process have a major bearing on deposition parameters, such as substrate temperature (and thereby, choice of substrate material) and throughput and coating properties, such as adhesion, crystallinity, grain size, lattice defects, transparency and surface roughness, and in general, the performance of the coating is inextricably linked to the choice of deposition process. The production of photoactive titania coatings is further complicated by the requirement for the coating to be predominantly in the anatase crystal form (mixed phase anatase/rutile structures have also been reported as being effective [10,20]). Titania coatings deposited at ambient temperature tend to be amorphous [12], though and the formation of anatase structures usually requires elevated temperatures (~400 °C) during deposition or post-deposition annealing, which imposes additional processing costs and restricts the use of thermally sensitive substrate materials.

Of the deposition techniques available, magnetron sputtering is widely used for the production of high quality coatings for applications ranging from Low-E and solar control glazing products, tool coatings, micro- and opto-electronic components, data storage media and thin film photovoltaics. Indeed, the scalability and versatility of the magnetron sputtering process and the uniformity and repeatability of the resulting coatings has made this the process of choice for many commercial applications [21].

The magnetron sputtering process has been described in detail elsewhere [21] and the finer nuances of magnetron design and process control are beyond the scope of this paper. In simple terms, though, it is a physical vapour deposition process in which positively charged ions from a glow discharge plasma are accelerated towards a negatively biased target plate of the material to be deposited, which is mounted on the magnetron body. The incident ions remove or “sputter” atoms from the surface of the target through a momentum exchange mechanism. The process takes place in a reduced pressure (typically 0.1 to 0.5 Pa) atmosphere, usually of argon, in which the plasma can be readily maintained. The sputtered atoms diffuse across the chamber and condense on the substrate as a thin film. Reactive gases, such as oxygen or nitrogen can be introduced with the argon in order to form compound films of oxides or nitrides. However, during the deposition of dielectric materials, such as oxides, the build-up of positive charges on the target can result in arc events, which are detrimental to the stability of the process and the quality of the coating. This problem can be negated by powering the magnetron in the mid-frequency (20–350 kHz) pulsed DC mode, where the polarity of the target alternates rapidly between positive and negative voltages. Again, this process has been described elsewhere [22].

Another variant of pulsed sputtering is the recently introduced HiPIMS (high power impulse magnetron sputtering) technique, which utilises lower pulse frequencies (50–1000 Hz), higher peak voltages (−500 to −1000 V) and very high peak currents (up to 1000 A). This results in similar time-averaged powers, but at much lower duty cycles, compared to pulsed DC magnetron sputtering, giving very high current densities at the target and leading to significant ionisation of the deposition flux. HiPIMS has been reported to enhance the film structure and make possible the deposition of crystalline thin films, including titania, without additional heat treatment [23,24]. Furthermore, the present authors have demonstrated that the thermal energy flux delivered to the substrate during HiPIMS deposition is several times lower than for DC or pulsed DC magnetron sputtering at the same time-averaged power [25]. This work was extended to demonstrate for the first time that photocatalytically active titania coatings can be deposited directly onto polymeric substrates by HiPIMS in a single stage process [26].

Sputtering systems can be configured with multiple magnetrons fitted with different target materials in order to deposit doped coatings, in which the dopant level is controlled by the relative power delivered to each magnetron. Alternatively, in a single magnetron system an alloy target can be used to produce doped coatings directly, although the dopant level in this case is fixed to that of the target material.

The ability to produce doped coatings is of great importance in this context, because the relatively high band gap of anatase (3.2 eV) means that it requires UV light (<390 nm) for activation. Photocatalytic activity can be both increased and extended into the visible range, though, by doping with different metallic elements (e.g., W, Mo, Nb, Ta) or non-metallic elements (e.g., N, C, S). Doping titanium dioxide with non-metal atoms narrows the band gap due to a mixing of the dopant p-states with the p-states of oxygen forming the valence band of titanium dioxide [27]. Of the range of possible non-metal dopants, nitrogen is one of the most described in literature for improving the photocatalytic activity of titanium dioxide [28,29,30] and extending its activity into the visible range. The nitrogen atom has a size comparable with the size of an oxygen atom, thus it can be easily introduced into the titania structure in either substitutional or interstitial positions [31].

Doping with transition metal ions is reported to create impurity levels near the conduction band that may perform as trapping centres, which extend the lifetime of photogenerated electrons and holes [32]. It is reported that the best results for transition metal doping can be achieved when the ionic radius of the doping metal is close to that of titanium [33] to enable incorporation into the titania lattice. Of the variety of candidate metals described in the literature, transition metals such as tungsten [34], chromium [35], vanadium [36] and molybdenum [37] are mentioned as efficient dopants for shifting the activity to the visible range.

Both of these doping strategies, and the idea of simultaneously co-doping titania with metallic and non-metallic elements, have been extensively investigated by many researches in the past few years [32,38]. Despite this, at present, there is no uniform theory explaining the optimum choice of dopant element(s) and doping level to maximise the photocatalytic properties in the visible range. This paper gives an overview of studies of doping and co-doping strategies conducted by the authors on magnetron sputtered titania coatings [25,39,40,41,42,43]. The influence of different elements on structural formation is considered and the production of as-deposited anatase coatings using the HiPIMS process is also described. Attempts to optimise the photoactivity of the coatings under UV, fluorescent and visible light irradiation are discussed.

## 2. Experimental Section

### 2.1. Coating Deposition Process

**Figure 1:**
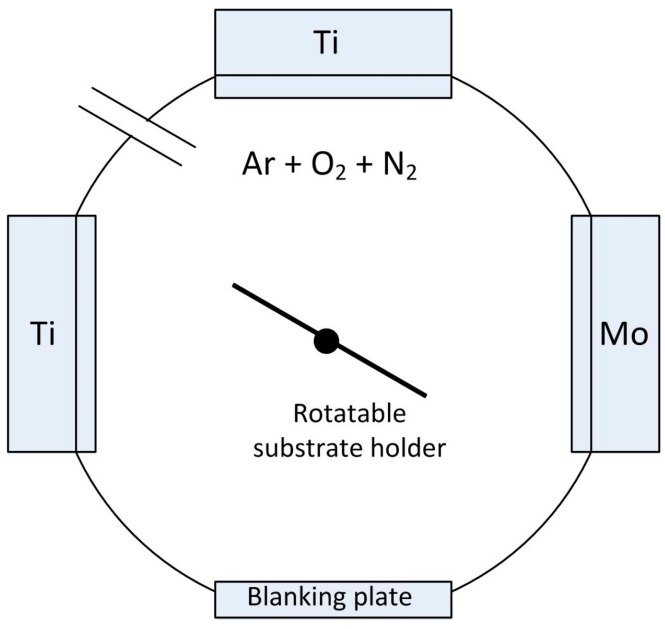
Schematic representation of the Teer Coatings Ltd. UDP450 sputtering rig with three planar magnetrons installed.

All the coatings described here were deposited by reactive magnetron sputtering in a Teer Coatings Ltd. (Droitwich, UK) UDP 450 system (Figure 1). Up to three 300 mm × 100 mm unbalanced planar magnetrons were installed vertically opposed through the chamber walls. Depending on the experimental array the system was configured with either two magnetrons fitted with titanium targets (99.5% purity) and one with a metallic dopant target (W, Mo, Ta or Nb—all 99.9% purity) [39,40,41], or for the HiPIMS array, a single magnetron was used with either a titanium target or a 5 at% W-doped Ti target installed [25,42,43].

For the multiple magnetron configuration, the magnetrons with the titanium targets were driven in mid-frequency pulsed DC mode using a dual channel Advanced Energy Pinnacle Plus supply at a frequency of 100 kHz and a duty of 50% (in synchronous mode) at a constant time-averaged power of 1 kW per channel. In order to vary the doping level, the magnetron with the dopant target was driven at powers in the range 100–180 W in continuous DC mode using an Advanced Energy MDX power supply. The reactive sputtering process was carried out in an argon:oxygen atmosphere at 0.3 Pa, and was controlled by optical emissions monitoring using an operating set point (15% of the full metal signal) previously found to produce stoichiometric TiO_2_ coatings [44]. The substrates (microscope slides initially, but later 20 × 10 mm^2^ 304 2B stainless steel coupons were also coated for antimicrobial testing) were ultrasonically pre-cleaned in propanol and placed onto the electrically floating substrate holder, which was rotated continuously during the deposition process at 4 rpm at a distance of 100 mm from the magnetrons. During the nitrogen and co-doping experiments, the nitrogen flow was controlled using a mass flow controller in the range from 0 to 10 sccm to vary dopant levels [41]. Coating thicknesses were in the range 500 nm to 1 µm. Initial experiments showed that the as-deposited pulsed DC coatings were amorphous. Therefore, these coatings were post-deposition annealed in air at either 400 or 600 °C for 30 min and then allowed to cool in air.

For the HiPIMS experiments, the magnetron was driven at time-averaged powers of 600 W and 880 W using a Huettinger HMP1/1_P2 HiPIMS power supply. The working pressure was varied in the range of 0.13 to 0.93 Pa. Pulse frequency (100–300 Hz) and pulse width (50–200 μs) were used as two other process variables. Sputtering was carried out in an argon:oxygen atmosphere of 2:3 for all deposition runs (10 sccm of Ar and 15 sccm of O_2_), which corresponded to the poisoned mode for this system. The thresholds of these variable parameters were chosen to maintain stable plasma discharge conditions and, thereby, control over the deposition process. The coatings were initially deposited onto soda-lime glass substrates. Coating thickness measurements were obtained by means of surface profilometry. All coatings deposited in this mode were of the order of 100 nm. Optimised operating conditions were then used to deposit coatings onto 100 µm PET (polyethylene terephthalate) web and PC (polycarbonate) substrates. The HiPIMS coatings were analysed in the as-deposited condition and were not annealed.

### 2.2. Coating Characterization

The coatings were typically analyzed by Raman spectroscopy (Renishaw Invia, 514 nm laser) and X-ray diffraction (XRD) in θ–2θ mode (Philips PW1729 diffractometer with CuKα1 radiation at 0.154 nm) to ascertain their crystalline structure. Composition was investigated by energy dispersive X-ray spectroscopy (EDX—Edax Trident, installed on a Zeiss Supra 40 VP-FEG-SEM). The surface roughness and surface areas of the coatings were determined using a MicroXAM white light surface profilometer. Finally, values of the optical band gaps of the coatings were calculated using the Tauc plot method [45], by plotting (α*hν*)^1/2^ *vs.* *hν* and extrapolating the linear region to the abscissa (where α is absorbance coefficient, *h* is Plank’s constant, *ν* is the frequency of vibration).

### 2.3. Assessment of Photocatalytic Activity and Hydrophilicity

The determination of photocatalytic activity was carried out using the methylene blue (MB) degradation test. MB is an organic dye with molecular formula C_16_H_18_ClN_3_S, and is often used as an indicating organic compound to measure the activity of photocatalysts. In fact, ISO10678 confirms the use of methylene blue as a model dye for surface photocatalytic activity determination in aqueous medium [46].

An aqueous solution of MB shows strong optical absorption at approximately 665 nm wavelength. Changes in the absorption peak height are used for monitoring the concentration of MB, and hence its degradation in contact with a photocatalytic surface.

Prior to the photocatalytic measurements, coating samples of equal size (15 × 25 mm^2^) were immersed in a conditioning solution of methylene blue for pre-absorption of MB on the test surfaces to exclude the effect of absorption during the photocatalytic experiment. The photocatalytic measurements were carried out for 1 h in continuous mode. The absorption peak height of the methylene blue solution was measured with an Ocean Optics USB 2000+ spectrometer with continuous magnetic stirring.

**Figure 2:**
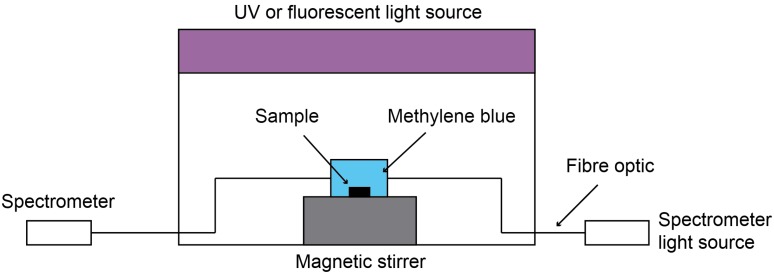
Schematic of methylene blue photocatalytic testing equipment.

Each coating was tested both under UV and fluorescent light sources; 2 × 15 W 352 nm Sankyo Denki BLB lamps were used as the UV light source (integrated power flux to the sample = 4 mW/cm^2^) and 2 × 15 W Ushio fluorescent lamps as the fluorescent light source (integrated power flux to the sample = 6.4 mW/cm^2^). Selected coatings were additionally tested under a visible light source. The visible light source was simulated by combining a fluorescent light source with a Knight Optical 395 nm long pass UV filter. The natural decay rate of methylene blue (without the photocatalyst present) under each type of light source was measured for reference purposes, as well as the degradation rate of methylene blue in contact with photocatalytic surface but without light irradiation (*i.e.*, in the dark). In both cases the decay rate of methylene blue was of zero order and, thus was neglected in the following calculations, meaning any changes in the absorption peak height could be attributed to the photocatalytic activity [37]. The experimental setup for the MB tests is shown schematically in Figure 2.

According to the Lambert-Beer law, the concentration of dye, *c*, is proportional to the absorbance value:

*A* = ε *cl* (1)

where *A* is absorbance, ε is the molar absorbance coefficient; *l* is the optical length of the cell where the photocatalyst is immersed into MB.

The photocatalytic decomposition of MB was approximated to first order kinetics, as shown in the equation:

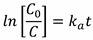
(2)
where *C*_0_ and *C* are the concentrations of MB solution at time 0 and time *t* of the experiment, respectively. If the ratio of absorption decay is proportional to the concentration decay, the first order reaction constant, *k_a_* can be found from the slope of the plot *ln*(*A*_0_/*A*) against time.

The hydrophilic properties of the coatings were estimated via measurements of contact angles of deionised water droplets on the surface of the coating made with a Kruss goniometer.

### 2.4. Assessment of Antimicrobial Properties

*Escherichia coli* (ATCC 8739) was used as a model organism in these experiments. Measurements of the antimicrobial activity of selected coatings deposited onto 304 2B stainless steel substrates were performed using ISO 27447:2009 as guidance (with minor modifications) [47]. Stainless steel was selected because it is the material of choice in the food and beverage production industries and the coatings were developed for field trials in industrial facilities [48,49]. In brief, 50 µL of suspension containing approximately 10^5^ colony forming units (cfu) per mL of bacterial cells were placed on the surfaces and a polyethylene film was placed over the bacterial suspension to ensure even distribution. Surfaces were illuminated (wavelength range of 300–700 nm) in a 20 °C incubator (Gallenkamp, Loughborough, UK) fitted with six fluorescent lamps (Sylvania, ON, Canada) with an energy output of 6.4 mW/cm^2^. At selected time points (0, 12, 24 and 48 h), surfaces were removed and vortexed for 1 min in neutralizing broth (20 g·L^−1^ Soya Lectin (Holland and Barrett, Nuneaton, UK) and 30 g·L^−1^ Tween 80 (Sigma Aldrich, Gillingham, UK) to remove any surviving bacteria. Bacteria were enumerated by plate counts. All tests were carried out in triplicate. Stainless steel was used as a light control and a set of coated surfaces were also kept in dark conditions to serve as further controls.

## 3. Results

### 3.1. Transition Metal-Doped Pulsed DC Coatings

#### 3.1.1. Structures and Compositions

**Figure 3:**
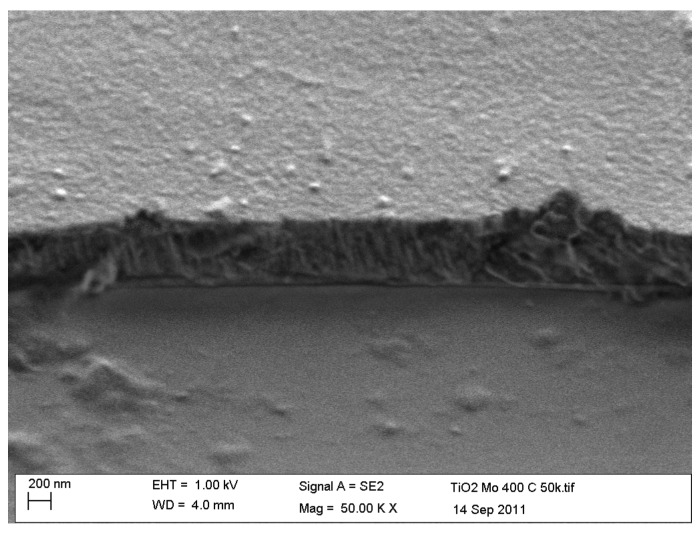
SEM micrograph of the fracture section of 2.44 at% Mo-doped titania coating deposited onto a glass substrate.

The coatings produced by pulsed DC sputtering had dense, defect-free structures, with relatively smooth surfaces. A typical example is shown in Figure 3, which is a SEM micrograph showing the fracture section and surface topography of a Mo-doped (2.44 at%) coating after annealing at 400 °C. The sputtering rates of the dopant metals investigated increased in order Nb < Mo < Ta < W. Thus, the dopant content increased in this order when the same given power was applied to the dopant target (see Table 1), meaning some calibration of the process is required if coatings with the same dopant content are required. However, that was not the overriding concern with these experiments, which were more focused on structural formation and photocatalytic activity.

**Table 1:** Dopant levels and band gap values for transition metal-doped titania coatings deposited on glass substrates and annealed.

Dopant	Power on Dopant Target	Dopant Content	Coating Thickness	Annealing Temperature	Band Gap	*k_a_* × 10^−5^, s^−1^	*k_a_* × 10^−5^, s^−1^	Crystal Structure
	W	at%	nm	°C	eV	UV light	fluor. light	
**none (pure TiO_2_**)	-	-	586	400	3.12	1.0	0.5	anatase
				600	3.15	1.7	0.6	anatase
**Nb**	100	0.74	607	400	3.16	2.0	0.5	anatase
				600	3.16	0.6	0.3	anatase
	150	1.94	697	400	3.15	1.8	0.5	anatase
				600	3.15	0.6	0.4	anatase
	180	2.67	712	400	3.13	1.5	0.9	anatase
				600	3.13	0.4	0.0	anatase
**Mo**	100	2.44	685	400	3.17	4.0	1.9	anatase
				600	3.00	2.8	1.8	anatase
	150	5.37	727	400	3.11	0.6	0.3	anatase
				600 *	2.95	-	-	anatase
	180	6.96	754	400	3.09	0.5	0.2	amorphous
				600 *	2.97	-	-	anatase
**W**	100	10.03	814	400	3.22	0.6	0.4	amorphous
				600	3.02	2.2	1.6	anatase
	150	13.87	889	400	3.22	0.6	0.7	amorphous
				600	3.00	1.4	0.8	anatase/rutile
	180	15.84	896	400	3.22	0.4	0.3	amorphous
				600	2.98	0.9	0.6	rutile
**Ta**	100	3.07	594	400	3.08	0.6	0.4	anatase
				600	3.09	1.3	0.6	anatase
	150	9.10	786	400	3.20	0.7	0.0	amorphous
				600	3.16	1.0	0.0	anatase
	180	13.51	943	400	3.28	0.9	0.0	amorphous
				600	3.24	0.7	0.0	rutile

* coatings delaminated from substrate and were not tested.

**Figure 4:**
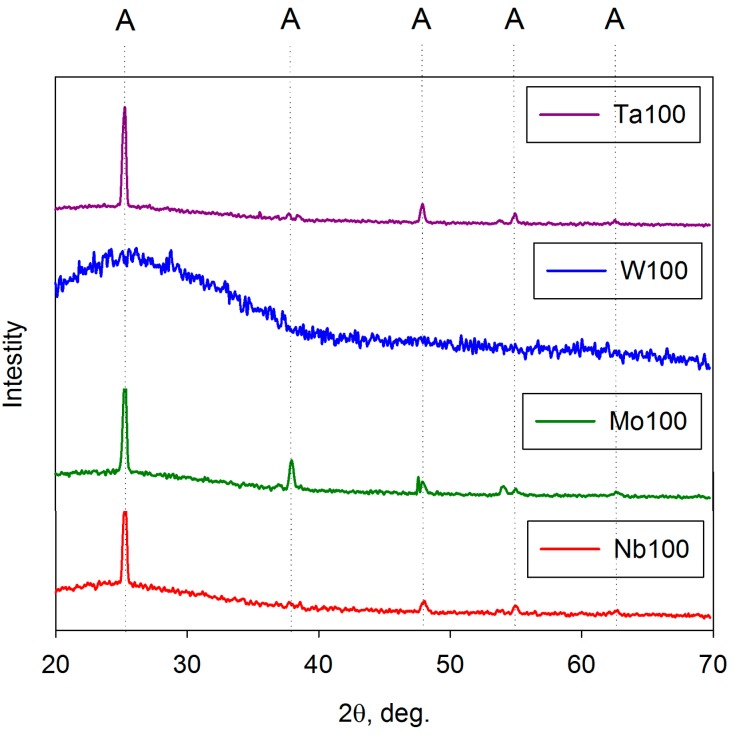
XRD analysis of selected doped-titania coatings deposited on glass substrates and annealed at 400 °C.

**Figure 5:**
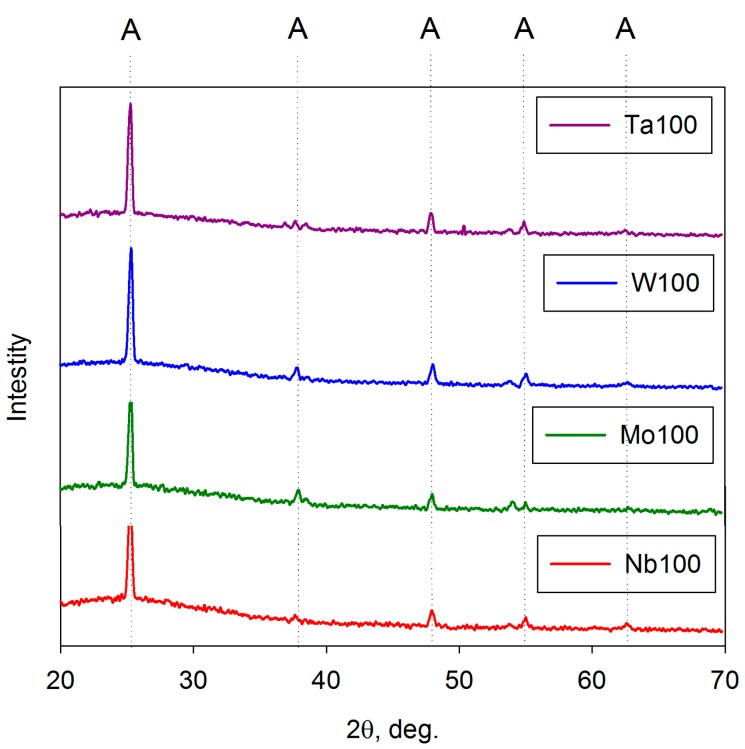
XRD analysis of selected doped titania coatings on glass substrates and annealed at 600 °C.

**Figure 6:**
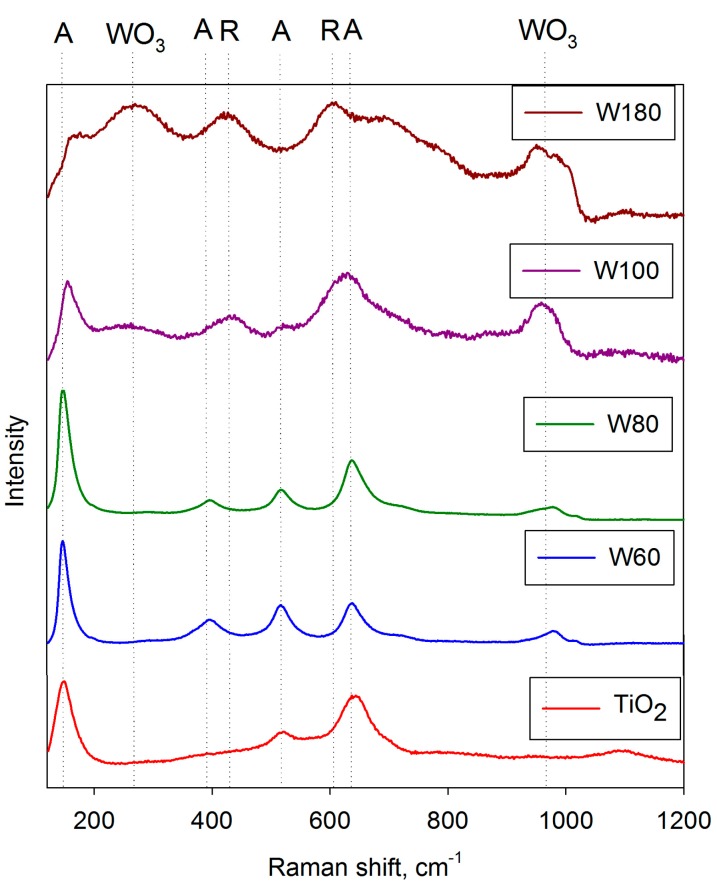
Raman spectra showing structural variations as a function of W-content for W-doped titania coatings after annealing at 600 °C.

As mentioned above, the as-deposited coatings were assumed to be amorphous on the basis of analysis by XRD and Raman spectroscopy. This concurs with previous work, which showed that for pure titania coatings, strongly crystalline anatase structures formed for coatings annealed at 400 °C and that this structure persisted up to 600 °C before evidence of rutile was observed [50]. For doped titania coatings, the dopant element has an important influence on structural formation during the annealing of these coatings. This is illustrated in Figure 4 and Figure 5, which show XRD spectra of selected doped-titania coatings annealed at 400 and 600 °C, respectively. The dopant compositions are indicated in Table 1. For Mo-, Ta- and Nb- doped coatings, a strong anatase structure has clearly evolved at 400 °C, whereas, doping with W appears to suppress the formation of this structure. Annealing at 600 °C results in the formation of an anatase structure for all the dopants investigated, but in the case of tungsten, broad rutile peaks were also detected in the Raman spectra for these samples (Figure 6), indicating a mixed-phase structure. This finding also highlights the different sensitivities of Raman spectroscopy and XRD for thin film analysis.

The choice of dopant material and annealing temperature also influenced the band gap of the resulting coating. Again, some typical examples are given in Table 1. Annealing at 400 °C produced very small red shifts for Mo-doped coatings, but small blue shifts for the other dopants. In contrast, annealing at 600 °C resulted in more significant red shifts for most of the combinations tested, and particularly for the Mo- and W- doped coatings (up to 0.2 eV).

#### 3.1.2. Photocatalytic Activity

As a benchmark for the doped titania coatings, the rate constants for the decomposition of methylene blue for pure titania coatings annealed at 400 °C and 600 °C sources were 1.0 × 10^−5^·s^−1^ and 1.7 × 10^−5^·s^−1^ under UV radiation and 0.5 × 10^−5^·s^−1^, and 0.6 × 10^−5^·s^−1^ under fluorescent light, respectively. As might be expected from the structural data shown in Figure 4, for the coatings annealed at 400 °C, Nb-doped coatings (best result: *k_a_* = 2.0 × 10^−5^·s^−1^ with 0.7 at% Nb) and Mo-doped coatings (best result: *k_a_* = 4.0 × 10^−5^·s^−1^ at 2.4 at% Mo) proved most effective at increasing photocatalytic activity under UV radiation. Only the 2.4 at% Mo-doped coating showed any notable improvement in fluorescent light activity (*k_a_* = 2.8 × 10^−5^·s^−1^), which again would be expected from the observed band gap shifts. Ta- and W- doped coatings showed a reduction in activity under both light sources.

For the coatings annealed at 600 °C, now both Nb and Ta proved ineffective as dopant elements, with reduced activities compared to pure titania. In this case, Mo-doped coatings and W-doped coatings showed the greatest increases in activity. The best rate constants obtained with 2.4 at% Mo were 2.8 × 10^−5^·s^−1^ and 1.8 × 10^−5^·s^−1^ for UV and fluorescent light radiation, respectively. The equivalent values for coatings with 10.0 at% W were 2.2 × 10^−5^·s^−1^ and 1.6 × 10^−5^·s^−1^.

#### 3.1.3. Optimisation of Tungsten Dopant Level

Although the W-doped coatings showed enhanced activity, it was recognised that the initial experimental conditions had produced relatively high levels of tungsten in the coatings (10–15 at%). Thus, a second series of W-doped coatings were produced where the power to the dopant target was varied over a lower range of values (60–90 W), to produce lower W dopant levels, with a view to optimising the activity level.

Other than the range of dopant target powers, the additional W-doped coatings were deposited under identical conditions to the initial batch of coatings, as described in Section 2.1. The coatings were then annealed at 600 °C. The dopant content and thickness of these and the previous W-doped coatings are given in Table 2. After annealing, these coatings showed a transition from anatase structures at low-W levels, through a mixed phase structure to rutile structures at higher W levels. Evidence for the formation of tungsten oxides was also identified for the higher W dopant levels. This structural transition with dopant content is illustrated in Figure 6, which shows selected Raman spectra for these coatings.

**Table 2:** Compositional data, band gap values and photocatalytic activity results for tungsten-doped titania coatings deposited on glass substrates and annealed at 600 °C.

Sample ID	Power on Dopant Target	Dopant Content	Coating Thickness	Band Gap	*k_a_* × 10^−5^, s^−1^	*k_a_* × 10^−5^, s^−1^	Crystal Structure
	W	at%	nm	eV	UV light	fluor. light	
**TiO_2_**	-	-	586	3.15	1.7	0.6	anatase
**W60**	60	3.83	702	3.12	4.0	1.0	anatase
**W70**	70	4.64	746	3.09	5.6	1.2	anatase
**W80**	80	5.89	758	3.09	9.9	2.7	anatase
**W90**	90	7.09	793	3.05	6.4	2.1	anatase
**W100**	100	10.03	814	3.02	2.2	1.6	anatase
**W150**	150	13.87	889	3.00	1.4	0.8	anatase/rutile
**W180**	180	15.84	896	2.98	0.9	0.6	rutile

**Figure 7:**
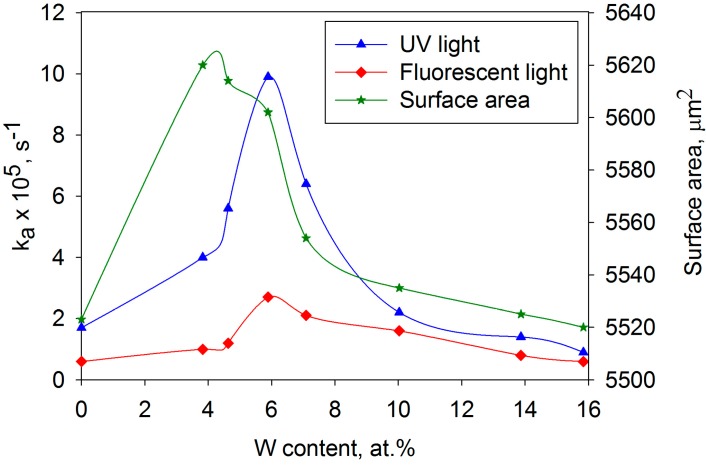
Variation in MB degradation rates for UV and fluorescent light irradiation and surface area, as functions of W content for W-doped titania coatings after annealing at 600 °C.

Once again, photocatalytic activity was assessed in terms of the degradation rate of methylene blue and band gap shifts were calculated from Tauc plots [40]. The results are also included in Table 2, together with surface area measurements calculated from white light profilometer scans. The rate constants obtained from the MB tests under UV and fluorescent light sources are also shown graphically as a function of W content in Figure 7, together with the surface area values for both sets of coatings. A clear, sharp peak in activity occurred at 5.9 at% W, with the *k_a_* values showing an approximately five-fold increase compared to the values for pure titania coatings. Further increases in W content beyond 5.9 at% lead to a rapid fall off in activity levels. The peak in activity appears to almost coincide with the peak in surface area of these coatings (as determined by white light profilometry). Whilst increased surface area would be expected to contribute to an increase in activity, due to the greater area in contact with the MB, consideration of the data presented shows that the maximum to minimum variation in surface area is only around 2%, which cannot alone account for the 500% increase in activity. Furthermore, the band gap of the coatings decreased progressively with W-content, from 3.12 at 3.8 at% to 2.98 at 15.8 at%, implying that the increased activity is not linked in this instance to a reduction in band gap energy.

A mechanism has been forward by a number of authors to account for the increase in activity at specific tungsten dopant levels [51]. When the photocatalyst is irradiated, the photogenerated electrons will be transferred into the tungsten oxide conduction band, which is located lower than the corresponding band of titanium dioxide (2.5–2.8 eV). Conversely, the holes will accumulate on the valence band of titania, promoting efficient charge separation. In the case of coatings with higher W content, excessive levels of dopant act as recombination centres for photogenerated electrons and holes. Additionally, the formation of a separate phase of tungsten oxide reduces the surface area of titanium dioxide, as proved by the surface morphology results, and thus reduces the area of contact between the pollutant and the photocatalyst. These factors result in a significant loss of photocatalytic activity for higher W-content coatings.

#### 3.1.4. Synergistic Effects of Co-Doping with Molybdenum and Nitrogen

To investigate the potential of co-doping with two elements, a batch of Mo-doped coatings were produced, which also incorporated varying levels of nitrogen. Coatings with the previously determined optimum Mo dopant level of 2.4 at% were used for these experiments. Coatings doped only with nitrogen were also produced for comparison purposes. Details of the coating compositions are given in Table 3. Once again, these coatings were post-deposition annealed at 600 °C prior to testing. Despite using the same range of flow rates, it can be seen that the nitrogen content was significantly lower in the N-doped only coatings, compared to the co-doped coatings. Indeed, the nitrogen content in coatings N1–N5 was too low to be quantified with the techniques used here (XPS and EDX). Co-doping with N and Mo is known to increase the solubility limits of both N and Mo in TiO_2_ [31]. This effect is described as being more pronounced in the case of nitrogen, as the solubility of N in titania is usually very low. The data presented here are in good agreement with this finding. Further detailed interpretation of the XPS analyses has been given elsewhere [41].

Raman analysis of the coatings confirmed an anatase structure for the annealed coatings, which XRD indicated had a strong (1 0 1) texture (not shown here) [41]. Band gap values and MB degradation rates are listed in Table 4. For the N-doped only coatings, samples N1 and N3 show some increase in UV activity, compared to the undoped titania coating, but apart from these two results, the effect of N-doping alone is negligible. However, the results for the co-doped coatings show a progressive increase in UV and fluorescent activity, with coating MoN7 demonstrating the highest activity under both light sources. This coating showed an increase in UV light activity of >4× and an increase in fluorescent light activity of >9× that of the pure titania coating. The equivalent values when compared to the Mo-only doped coating are both approximately a 3× increase in activity. Furthermore, visible light testing (using the 395 nm long pass filter) demonstrated that these coatings also exhibited some activity under this light source, while for undoped/N-doped titania coatings no activity was recorded.

**Table 3:** Compositional properties and thickness of titania coatings doped with nitrogen (“N” series) and co-doped with molybdenum/nitrogen (“MoN” series).

Dopant	Sample ID	Flow of Nitrogen, Sccm	Content of Nitrogen, at%	Coating Thickness, nm
Mo	TiO_2_ + Mo	-	-	685
N	N1	1	<1%	654
	N3	3	<1%	657
	N5	5	<1%	654
	N7	7	1.09	658
	N10	10	3.67	661
Mo + N	MoN1	1	1.22	760
	MoN3	3	3.08	764
	MoN5	5	4.95	758
	MoN7	7	7.13	761
	MoN10	10	9.12	766

**Table 4:** Band gap values and MB degradation rate constants for N-doped and Mo-N co-doped titania coatings.

Sample ID	Band Gap	Band Gap Shift (Compared to TiO_2_)	*k_a_* × 10^−5^, s^−1^	*k_a_* × 10^−5^, s^−1^	*k_a_* × 10^−5^, s^−1^
	eV		UV Light	Fluor. Light	Vis. Light
TiO_2_	3.15	-	1.7	0.6	0
TiO_2_ + Mo	3.00	−0.15	2.8	1.8	0.6
N1	3.22	+0.07	3.6	0.9	0
N3	3.22	+0.07	2.9	1.6	0
N5	3.20	+0.05	1.7	1.1	0
N7	3.14	−0.01	1.7	1.0	0
N10	3.08	−0.07	1.4	0.9	0
MoN1	3.09	−0.06	1.0	0.6	0
MoN3	3.09	−0.06	4.9	1.7	0.4
MoN5	3.05	−0.10	6.9	2.1	0.7
MoN7	3.04	−0.11	7.5	5.6	1.2
MoN10	3.07	−0.08	5.6	3.7	1.0

The results of photocatalytic tests showed that doping with nitrogen only had at best a moderately positive effect on photocatalytic activity, while co-doping with nitrogen and molybdenum resulted in significant improvements in photocatalytic activity. The efficiency of N-doped coatings under UV light, compared to that of undoped titania, was higher by a factor of 2 at most and generally lower than this. However, despite widely published information about N-doping as an efficient method of improving the photocatalytic properties under fluorescent/visible light [30,52], the N-doped titania coatings studied in this work had only a marginally higher efficiency of MB degradation under the fluorescent light source. As no noticeable band gap shift towards the visible range was observed, the increased photocatalytic activity under the fluorescent light source could only be attributed to improved electron-hole separation and the extended lifetime of charge carriers, as a result of nitrogen incorporation.

The observed increase in activity of the co-doped coatings can be assumed to be a result of more efficient electron-hole separation, compared to undoped or singly Mo- or N-doped titania coatings, due to the synergistic effect of Mo-N co-doping. A mechanism of explaining more efficient charge carrier separation was proposed by Cheng *et al.*, who observed similar results for Mo-N co-doped coatings prepared by a hydrolysis-precipitation method [38]. In the proposed mechanism nitrogen and molybdenum create local energy levels within the titania band gap, and therefore several ways of charge carrier excitation are available, and consequently more photo-induced charge carriers can be efficiently separated to participate in the photocatalytic process. Co-doped coatings with optimum content of nitrogen and molybdenum demonstrate significantly higher photocatalytic activity, due to more efficient charge carrier separation and their extended lifetimes. A shift of the band gap towards the visible range, compared to undoped titania, enables the presence of photocatalytic activity under fluorescent and visible light sources.

### 3.2. Characterisation of As-Deposited HiPIMS Titania Coatings

**Figure 8:**
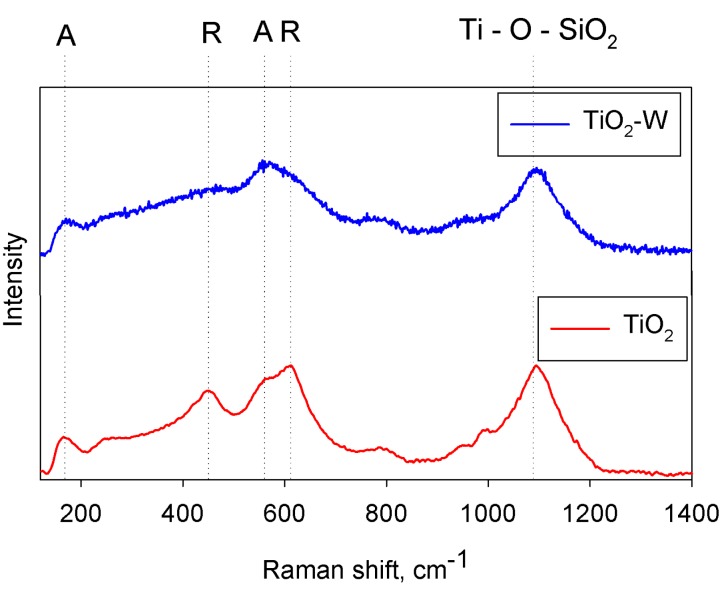
Raman spectra of as-deposited W-doped (TiO_2_-W) and undoped titania (TiO_2_) coatings deposited by HiPIMS on glass substrates.

In a preliminary study using the HiPIMS process, coatings were deposited from a single titanium target and a Ti target containing 5 at% W [42]. Analysis by Raman spectroscopy of the as-deposited coatings indicated that the pure titania samples had formed a mixed anatase/rutile structure, whereas the W-doped coatings had only a weakly crystalline anatase structure. Example spectra are shown in Figure 8. These spectra imply that the presence of tungsten in the coating has suppressed the formation of a crystalline structure, as observed previously for the coatings deposited by pulsed DC sputtering. The band gap data and photocatalytic activity rate constants for these coatings are listed in Table 5. A number of interesting points emerge from these data. Firstly, the UV light rate constants (*k_a_* values ~2.0 to 2.4 × 10^−5^·s^−1^) are noticeably higher than those obtained for pulsed DC titania coatings after annealing (typically *k_a_* = 1.7 × 10^−5^·s^−1^). It was also observed that the presence of W reduced the band gap of these coatings quite considerably (by 0.14–0.15 eV), which in turn lead to a 2 to 4 fold improvement in the level of fluorescent light activity for the doped coatings. Finally, the W-doped HiPIMS titania coatings displayed visible light activity levels very close to the values measured under fluorescent light sources.

**Table 5:** Overview of deposition conditions and properties of titania and W-doped titania coatings deposited on glass substrates by HiPIMS.

Coating ID	Mean Sputtering Power	W	Band Gap	Surface Roughness	Surface Area	*k_a_* × 10^−5^	*k_a_* × 10^−5^	*k_a_* × 10^−5^
	kW	at%	eV	μm	μm^2^	UV Light, s^−1^	Fluorescent Light, s^−1^	Visible Light, s^−1^
TiO_2_	44	-	3.21	0.084	5521	2.0	0.3	-
TiO_2_W	44	5.67	3.04	0.092	5524	2.1	1.2	0.9

**Figure 9:**
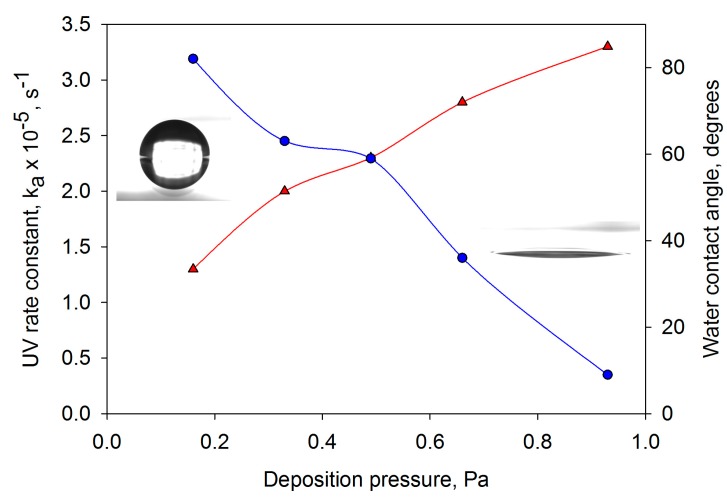
Variation in water contact angle and UV light photocatalytic rate constant as a function of deposition pressure for titania coatings deposited on glass substrates by HiPIMS. Images of the water droplets are included for samples deposited at 0.16 and 0.93 Pa.

Having demonstrated the potential of the HiPIMS process, further studies were carried out to attempt to optimise the performance of the coatings produced by this technique [43]. Process variables including deposition pressure, pulse width (*i.e.*, duration of the power pulse delivered to the target) and pulse frequency were varied and their impact on photocatalytic activity and water contact angle was investigated. Of these variables, deposition pressure emerged as the most influential. This is clearly illustrated in Figure 9, which compares the variation with coating pressure of contact angle and the photocatalytic activity rate constants for MB degradation under UV light irradiation. Following these experiments, coatings were deposited directly onto PET and polycarbonate (PC) substrates under optimised conditions. Within experimental accuracy, the same values of first order rate constants were obtained for these coatings, independent of the substrate materials tested (glass, PET and PC).

### 3.3. Antimicrobial Activity

The antimicrobial activity against *E. coli* of selected Mo- and W- doped titania coatings was assessed and compared to pure titania coatings and stainless steel controls. As mentioned earlier, 304 2B stainless steel was chosen for its compatibility with industrial processing. The coatings were deposited by pulsed DC magnetron sputtering, using the conditions described earlier, and then annealed at 600 °C. Interestingly, for this choice of substrate material, a higher molybdenum content (6.9 at%) was found to provide the greatest photocatalytic activity from MB tests (compared with 2.44 at% for glass), so this dopant content was used, along with coatings doped with 3.8 at% W, which were also found to be optimal in this case. This finding concurs with other studies that have shown that the choice of substrate material (particularly whether it is electrically conductive or not) influences photocatalytic activity [53].

**Figure 10:**
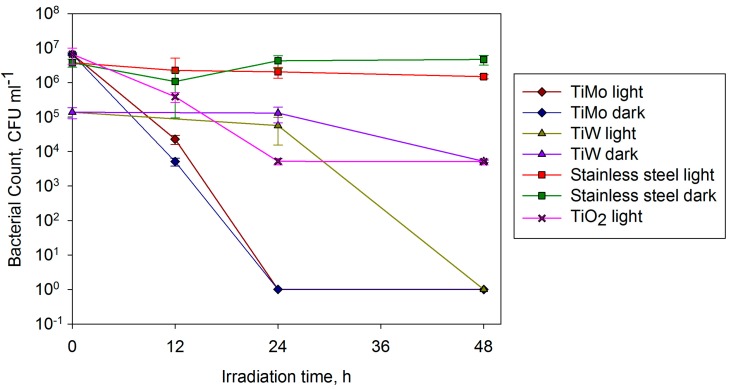
Antimicrobial effect of TiO_2_, TiO_2_-Mo and TiO_2_-W surfaces on *Escherichia coli* Stainless steel surfaces were used as controls and light and dark conditions were investigated.

The stainless steel controls did not reduce the number *E. coli* colony forming units in light or dark conditions in a 48 h period (Figure 10). The pure titania coatings showed only a weak photocatalytic effect in reducing the number of colony forming units by 2-logs in this time period. However, coatings doped with Mo eradicated *E. coli* within 24 h in both light and dark conditions. The activity displayed in the dark suggests that the surface is dual functioning, being both photocatalytic (as determined by the degradation of MB) and innately antimicrobial. W-doped coatings also reduced microbial counts by 5-logs within 48 h in the light but not the dark, *i.e.*, only photocatalytic behaviour was observed. All doped surfaces displayed an ability to inactivate *E. coli* when tested under visible light and, in the Mo case, when in the dark, highlighting the potential use of such surfaces for indoor applications, allowing a choice between a coating with an active antimicrobial function (Mo), or one which is inert unless irradiated (W), depending on requirements and regulations.

## 4. Discussion

**Figure 11:**
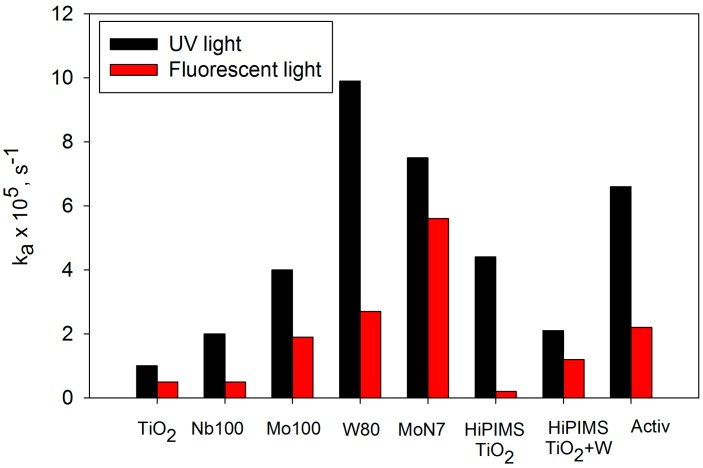
Bar chart of maximum rate constants obtained for the decomposition of methylene blue under UV and fluorescent light sources for pure titania coatings, transition metal doped titania coatings, Mo and N co-doped coatings deposited by pulsed DC magnetron sputtering (annealed at 600 °C) and titania and W-doped titania coatings deposited by HiPIMS (as-deposited).

This paper has considered a number of deposition and doping strategies for the production of titania-based photocatalytically active thin films. Reactive magnetron sputtering is a versatile, flexible technique for the production of high quality, fully dense coatings. When operating in pulsed DC mode, it provides a stable, arc-free process for the deposition of dielectric materials, such as titania. Furthermore, the coatings can be readily doped via transition metals, or via non-metal gaseous species, or a combination of both. In each case, control of the dopant level is straightforward. However, when operating in this mode, the as-deposited coatings were found to be amorphous and, therefore, showed no activity. Effective annealing temperatures for structural formation varied with dopant element. Mo-doped coatings annealed at 400 °C were found to demonstrate significantly higher activities than pure titania coatings annealed at the same temperature, whereas a temperature of 600 °C was required to achieve the same result for the W-doped coatings. Figure 11 additionally shows that the synergistic effect obtained by co-doping with Mo and N also produced coatings with a UV activity, close to that of the W-doped coatings, and a noticeably higher activity in fluorescent light. The UV and fluorescent light activities of the W-doped and MoN co-doped coatings also exceed the values shown in Figure 11 obtained for a sample of Pilkington’s Activ, which is a commercially available product. Direct comparisons with this sample should be avoided, because it is produced via a chemical vapour deposition pyrolysis route and is significantly thinner than the sputtered coatings. However, as there is a dearth of “standard samples” in this field, it still serves as a useful guide to relative activity levels. A mixed anatase/rutile phase was detected for the samples with the highest levels of tungsten doping in the pulsed DC study, although the best photocatalytic results were still found for anatase coatings. In contrast, for the pure titania deposited via HiPIMS, a mixed phase structure gave superior photocatalytic activity [43]. This, of course, is not a new finding and several researchers have proposed that the mixed phase structure is optimal for photocatalytic activity [10,20].

The HiPIMS process is still in the development stage and there remain issues with power supply stability and process control. Nevertheless, the potential of this process to produce, at least, semi-crystalline coatings in the as-deposited state is a clear advantage over other deposition processes. Furthermore, the low net deposition temperature makes it a suitable technique for deposition onto thermally sensitive materials, as demonstrated here with PET and PC substrates. The data presented in Figure 11 indicates that, when optimised, the HiPIMS pure titania coatings could achieve approximately twice the UV activity rate of the annealed pulsed DC coatings. The W-doped HiPIMS showed a reduction in UV activity, attributed to the weaker crystalline structure, but higher fluorescent light activity, attributed to a substantial band gap shift.

The capacity to break down organic compounds, as modelled here with methylene blue, is just one of the phenomena associated with photocatalytic coatings. The inactivation of microorganisms is another important ability. Numerous researchers have claimed antimicrobial activity for their coatings, but care must be taken in assessing these claims. The test method for antibacterial activity of photocatalytic materials is complex, requiring specific experimental conditions to be met and multiple repeat experiments if results are to be tested for reproducibility and compared to other published data. The results presented here are a case in point. A limited number of replicates were tested and only one microorganism was used; the Gram-negative *E. coli*. Ideally, more replicates would be tested and a Gram-positive microorganism, such as *Staphylococcus aureus*, would also be investigated. Despite this, the doped titania coatings showed the ability to eradicate *E coli* within 24 to 48 h. There was also an interesting distinction between the dopant elements, with the Mo-doped coatings being effective in light and dark and the W-doped coatings only being effective in the light. These results certainly merit more detailed investigation in the future. The recent introduction of antibacterial testing under indoor lighting (ISO 17094:2014) [54] has now allowed for visible light active photocatalytic surfaces to be tested more precisely, however, a more rapid antimicrobial testing method which could be performed by non-microbiologist would still be valuable.

## 5. Conclusions

Reactive magnetron sputtering techniques have been used to produce a range of titania and doped titania coatings. Choice of deposition technique (pulsed DC sputtering or HiPIMS) and choice of dopant element had a significant influence on structural formation and, subsequently, photocatalytic activity for these coatings. Pulsed DC coatings were amorphous in the as-deposited state, with no measurable activity against methylene blue, whereas the HiPIMS coatings were weakly crystalline as-deposited with moderate levels of activity. The benefits of this technique were further demonstrated by depositing active coatings onto polymeric substrates in a single stage process. Of the transition metals investigated as dopant elements, molybdenum and tungsten were the most effective. The highest UV activity recorded in these experiments was achieved by coatings doped with 5.9 at% W after annealing at 600 °C. This was slightly higher than the UV activity of MoN-doped coatings after annealing, but the co-doped coatings showed a higher level of activity under fluorescent light irradiation. Although only limited tests were performed, the Mo- and W- doped coatings also demonstrated the ability to inactivate *E. coli*. In the former case, the coatings were both antimicrobial (active in the dark) and photocatalytic (active in the light), whereas the W-doped coatings only displayed photocatalytic activity.
